# ﻿The redescription and complete mitogenomes of two *Oxycarenus* species (Hemiptera, Oxycarenidae) and phylogenetic implications

**DOI:** 10.3897/zookeys.1211.126013

**Published:** 2024-09-05

**Authors:** Changjun Meng, Suyan Cao, Wen Dong, Cuiqing Gao

**Affiliations:** 1 Co-Innovation Center for Sustainable Forestry in Southern China, College of Forestry and Grassland, Nanjing Forestry University, Nanjing, Jiangsu 210037, China Nanjing Forestry University Nanjing China

**Keywords:** Heteroptera, Lygaeoidea, mitochondrial DNA, *Oxycarenusbicolorheraldus*, *Oxycarenusgossypii*, phylogenetic analysis

## Abstract

In this study, the two Oxycarenidae species, *O.gossypii* Horváth, 1926 and *Oxycarenusbicolorheraldus* Distant, 1904, are redescribed, and their complete mitogenomes are sequenced and analyzed. The phylogeny of Lygaeoidea is examined using 45 complete mitogenomes of lygaeoid species and four outgroup species. The gene orientation and arrangement of the two mitogenomes are found to be consistent with typical Lygaeoidea mitochondrial features, comprising 37 genes, including 13 PCGs, 22 tRNAs, 2 rRNAs, and a control region. Nucleotide composition of the species was biased towards A and T, with the gene order identical to the putative ancestral arrangement of insects. Start codons, stop codons, RNAs, relative synonymous codon usage (RSCU), and nucleotide diversity (Pi) of Oxycarenidae exhibited characteristics similar to other families in Lygaeoidea. Bayesian-inference (BI) and maximum-likelihood (ML) methods were employed to investigate phylogenetic relationships using PCG datasets from selected species. Phylogenetic analyses reveal slightly different topologies between BI and ML methods, with variation primarily concentrated in Colobathristidae and Rhyparochromidae. Our study confirms that the two sequenced Oxycarenidae species formed a single clade, and the position of Oxycarenidae remains stable in both ML and BI phylogenetic trees. These findings expand the mitochondrial genome databases of Lygaeoidea and provide valuable insights into the phylogenetic relationships within Lygaeoidea or Pentatomomorpha.

## ﻿Introduction

Mitochondrial genome analysis is a powerful tool for elucidating the phylogeny and population genetics of insect taxa (Cameron and Whiting 2008; Li et al. 2023). Insects possess circular double-stranded mitochondrial molecules typically ranging from 14 to 20 kb in length. These genomes encode 37 genes, encompassing 13 protein-coding genes (PCGs), two ribosomal RNA genes (rRNAs), 22 transfer RNA genes (tRNAs), and a single control region. While insect mitochondria generally adhere to a conventional structure, there are exceptions, in certain species of Anoplura (Boore 1999; Shao et al. 2009). Characterized by compactness, insect mitochondrial genomes feature minimal spacer regions or overlapping sequences between adjacent genes (Boore 1999). Notably, they exhibit small size, stable genetic composition, relatively conserved gene sequences, rapid evolutionary rates, and comprehensive molecular information. Consequently, they serve as invaluable tools for investigating molecular evolution, phylogenetics, and population genetic structure (Xue and Bu 2008; Simon and Hadrys 2013; Kocher et al. 2014).

The Lygaeoidea, the second largest superfamily in Pentatomomorpha, comprises over 4,700 described species across 16 families (Weirauch and Schuh 2011; Dellapé and Henry 2024). This widespread terrestrial superfamily primarily includes herbivorous species feeding on plant seeds or sap, with some being economically significant pests (Sweet 2000). Among them, Oxycarenidae species predominantly inhabit the plants of Malvaceae and Sterculiaceae, where they feed on seeds and can inflict substantial damage to cotton and other mallow crops (Sureshan et al. 2021). Currently, over 140 species in 27 genera have been documented worldwide (Henry and Dellapé 2009; Xiao and Gao 2022; Dellapé and Henry 2024).

Past studies investigating the phylogenetic relationships of Pentatomomorpha have relied on morphological characters for classification (Henry 1997), and more recently, they have incorporated molecular data. The increasing number of Lygaeoidea species documented in recent years underscores the importance of exploring their phylogenetic relationships using mitochondrial DNA, both to validate previous findings and to provide additional insights.

In this study, we redescribe both *O.gossypii* Horváth, 1926 and *Oxycarenusbicolorheraldus* Distant, 1904. Additionally, two misidentifications in China are rectified, and the complete mitogenomes of these species are sequenced. Subsequently, we construct phylogenetic trees using the complete mitogenomes of 45 species of Lygaeoidea and four outgroup species. These findings contribute essential data for further investigations into the phylogenetic relationships within Lygaeoidea and Pentatomomorpha.

## ﻿Materials and methods

### ﻿Sample collection, identification and DNA extraction

Adult specimens of *Oxycarenusbicolorheraldus* Distant, 1904 were collected from Xiangshan Park, Pukou District, Nanjing, Jiangsu Province, China, in May 2020. Adult specimens of *O.gossypii* Horváth, 1926 were collected from Phoenix Airport, Sanya City, Hainan Province, China, in March 2020.

Composite images were obtained using an M205FA Leica stereomicroscope and camera, with the Leica Application Suite v. 4.5.0. Type label data are presented verbatim, with lines on the same label separated by a slash (/), and different labels divided by double slashes (//). Texts printed [pr] and handwritten [hw] are indicated. All measurements provided in the text are expressed in millimetres.

### ﻿Abbreviations

**BMNH**Natural History Museum, London, United Kingdom;

**IZAS**Institute of Zoology, Academia Sinica, Beijing, China;

**MSIE** Shanghai Institute of Entomology, Shanghai, China;

**NKUM**Institute of Entomology, Nankai University, Tianjin, China;

**NJFU**Nanjing Forestry University, Nanjing, Jiangsu.

Genomic DNA were extracted from adult target insects using the Rapid Animal Genomic DNA Isolation Kit (Sangon Biotech, Shanghai, China).

### ﻿Sequencing, assembly, annotation, and bioinformatics analyses

The mitochondrial genomes of these two species were sequenced using an Illumina MiSeq PE300 platform (Sangon Biotech, Shanghai, China). Subsequently, Fastp v. 0.36 (Chen et al. 2018) was employed to eliminate low-quality and short reads, ensuring the integrity of the data for subsequent analysis. SPAdes v. 3.15 (Bankevich et al. 2012) facilitated the de novo assembly of the high-quality next-generation sequencing data, resulting in the generation of contigs and scaffolds. Rigorous evaluation and quality control measures were applied to the assembly results using PrInSeS-G (Massouras et al. 2010). Potential contamination originating from the host genome was meticulously identified and eliminated, retaining only the scaffolds derived from the organelle genome. Sequence similarity was assessed by comparing the scaffolds with the NCBI library using BLASTn (Ye et al. 2012). Target scaffolds were manually selected based on sequencing depth and coverage information for each scaffold. GapFiller v. 1.11 (Boetzer and Pirovano 2012) was utilized to supplement and rectify obtained alleles by correcting editing errors and filling gaps introduced during splicing, including the insertion or deletion of fragments as needed.

The two mitogenome sequences were annotated using Geneious v. 11.0.2 (Kearse et al. 2012), following the invertebrate mitochondrial genetic code. Circular maps of the mitogenomes were generated using the CGView Server (Grant and Stothard 2008). To ensure annotation accuracy, all tRNA genes were verified using the MITOS Web Server (Bernt et al. 2013), and their secondary structures were predicted using the tRNAscan-SE Server v. 1.21 (Lowe and Chan 2016). PhyloSuite v. 1.2.3 (Xiang et al. 2023) and MEGA X (Kumar et al. 2018) were employed to determine base composition and relative synonymous codon usage (RSCU) values of the two mitogenome sequences. Non-synonymous substitutions (Ka) and synonymous substitutions (Ks) of the 13 PCGs of Oxycarenidae were calculated using DnaSP5 software (Librado and Rozas 2009), and Ka/Ks values were subsequently derived. Nucleotide composition skew was computed using the formulas developed by Perna and Kocher: AT-skew = (A − T) / (A + T) and GC-skew = (G − C) / (G + C). This study aimed to comprehensively examine the evolutionary patterns among mitochondrial protein-coding genes (PCGs) in species of Oxycarenidae.

### ﻿Phylogenetic analysis

To investigate mitogenome arrangement patterns in Lygaeoidea, we compared the gene orders of all known Lygaeoidea mitogenomes with those of closely related taxa (Table 1). For phylogenetic analyses, we examined a total of 49 mitogenomes (Table 1), which included two newly generated sequences from this study. We standardized all sequences and extracted 13 PCGs using PhyloSuite v. 1.2.2 (Perna and Kocher 1995; Xiang et al. 2023). The 13 PCGs of these species were individually aligned using codon-based multiple alignments with MAFFT v. 7.313 software (Katoh and Standley 2013). The concatenated PCGs were processed with PhyloSuite v. 1.2.3. PartitionFinder2 selected optimal partitioning schemes and evolutionary models for constructing Bayesian-inference (BI) and maximum-likelihood (ML) trees with confidence (Soria-Carrasco et al. 2007; Lanfear et al. 2017). Phylogenetic trees were reconstructed using IQ-TREE v. 1.6.8 (Guindon et al. 2010) and MrBayes v. 3.2.6 (Ronquist et al. 2012) with the assistance of PhyloSuite v. 1.2.2.

**Table 1.** Sequences used in this study.

Superfamily	Family	Species	Length (bp)	GenBank No.
Lygaeoidea	Berytidae	*Metatropislongirostris* Hsiao, 1974	15,744	NC_037373.1
	Berytidae	*Yemmalysusparallelus* Stusak, 1972	15,747	NC_012464.1
	Blissidae	*Bochrusfoveatus* Distant, 1879	14,738	ON961018.1
	Blissidae	*Capodemussinuatus* (Slater, Ashlock & Wilcox, 1969)	15,199	ON961019.1
	Blissidae	*Caveleriusyunnanensis* Gao & Zhou, 2021	15,330	NC_065816.1
	Blissidae	*Dimorphopterusgibbus* (Fabricius,1794)	14,988	NC_065817.1
	Blissidae	*Iphicratesgressitti* Slate, 1966	15,288	NC_065818.1
	Blissidae	*Ischnodemusnoctulus* Distant, 1901	15,291	NC_065819.1
	Blissidae	*Macropesdentipes* Motschulsky, 1859	14,923	NC_065821.1
	Blissidae	*Macropesharringtonae* Slater, Ashlock & Wilcox, 1969	15,314	OP442511.1
	Blissidae	*Macropesrobustus* Zheng & Zou, 1982	15,041	NC_065822.1
	Colobathristidae	*Phaenacanthamarcida* Horváth, 1914	14,540	NC_012460.1
	Geocoridae	*Geocorispallidipennis* (Costa, 1843)	14,592	NC_012424.1
	Geocoridae	*Henestarishalophilus* (Burmeister, 1835)	14,868	MW619656.1
	Lygaeidae	*Arocatusmelanocephalus* (Fabricius,1798)	15409	NC_063142.1
	Lygaeidae	*Crompusoculatus* Stål, 1874	15,332	MW619652.1
	Lygaeidae	*Kleidocerysresedaeresedae* (Panzer, 1793)	14,688	KJ584365.1
	Lygaeidae	*Lygaeus* sp. FS-2019	15,235	MF497725.1
	Lygaeidae	*Nysiuscymoides* (Spinola, 1837)	16,301	MW291653.1
	Lygaeidae	*Nysiusfuscovittatus* Barber, 1958	14,575	NC_050167.1
	Lygaeidae	*Nysiusgraminicola* (Kolenati, 1845)	16760	NC_073587.1
	Lygaeidae	*Nysiusplebeius* Distant, 1883	17,367	MN599979.1
	Lygaeidae	*Nysius* sp.	16,330	MW465654.1
	Lygaeidae	*Pylorgusporrectus* Zheng, Zou & Hsiao, 1979	15,174	NC_080509.1
	Lygaeidae	*Pylorgussordidus* Zheng, Zou & Hsiao, 1979	15,399	NC_084343.1
	Lygaeidae	*Tropidothoraxcruciger* (Motschulsky, 1859)	15,781	NC_056293.1
	Lygaeidae	*Tropidothoraxsinensis* (Reuter, 1888)	15,422	MW547017.1
	Malcidae	*Chauliopsfallax* Scott, 1874	15,739	NC_020772.1
	Malcidae	*Chauliops* sp.	15300	OP793778.1
	Malcidae	*Chauliopsquaternaria* Gao & Bu, 2009	15612	NC_087837.1
	Malcidae	*Chauliopszhengi* Xue & Bu, 2004	15507	NC_087838.1
	Malcidae	*Malcusauriculatus* Štys, 1967	15,097	NC_063141.1
	Malcidae	*Malcusinconspicuous* Štys, 1967	15,316	OL944394.1
	Malcidae	*Malcussetosus* Štys, 1967	14,894	NC_063138.1
	Ninidae	*Cymoninussechellensis* (Bergroth, 1893)	15,962	NC_085420.1
	Ninidae	*Ninusinsignis* Stål, 1860	14,632	NC_063137.1
	Oxycarenidae	*Oxycarenusgossypii* Horváth, 1926	16,144	OR_713903
	Oxycarenidae	*Oxycarenusbicolorheraldus* Distant, 1904	15,462	PP_446310
	Rhyparochromidae	*Bryanellocorisorientalis* Hidaka, 1962	15,606	NC_063139.1
	Rhyparochromidae	*Eucosmetusincises* (Walker, 1872)	14,562	NC_085565.1
	Rhyparochromidae	*Harmosticafulvicornis* (Horváth, 1914)	15,703	NC_063140.1
	Rhyparochromidae	*Ligyrocorissylvestris* (Linnaeus, 1758)	16,621	PP145295.1
	Rhyparochromidae	*Neolethaeusassamensis* (Distant, 1901)	15,067	NC_037375.1
	Rhyparochromidae	*Panaorusalbomaculatus* (Scott, 1874)	16,345	NC_031364.1
Pyrrhocoroidea	Pyrrhocoridae	*Dysdercusevanescens* Distant, 1902	15,635	MW619727.1
Coreoidea	Alydidae	*Riptortuspedestris* (Fabricius, 1775)	17,191	EU427344.1
	Coreidae	*Hydaropsislongirostris* (Hsiao, 1963)	16,521	EU427337.1
	Rhopalidae	*Aeschyntelusnotatus* Hsiao, 1963	14,532	EU427333.1

## ﻿Results

### ﻿Taxonomy

#### ﻿ Oxycarenus gossypii

Taxon classificationAnimaliaHemipteraOxycarenidae

Horváth, 1926

7CE1A317-74E0-5474-BBF9-5053F24D28B7

Figs 1A , 2A–C

Oxycarenus gossypii : Horváth 1926: 136; Esaki 1926: 161; Slater 1964: 673; Péricart 2001: 115.
Oxycarenus laetus : Zheng and Zou 1981: 96. Misidentification.

##### Material examined.

China • 3♂♂1♀; Yunnan, Yuanjiang; alt. 400 m; 25 Jul. 2006; Weibing Zhu leg. (NKUM) • 1♂2♀♀; Yunnan, Xishuangbanna, Mengsong; alt. 1600 m; 23 Apr. 1958; Xvwu Meng leg. (IZAS) • 1♂; Yunnan, Xishuangbanna, Damenglong; alt. 650 m; 8 Apr. 1958; Leyi Zheng leg. (IZAS) • 5♂♂6♀♀; Hainan, Sanya, Fenghuang airport; 26 Mar. 2020; Bo Cai leg. (NJFU) • 16♂♂5♀♀; Hainan, Jianfengling thermal forestry institute; 21 Apr. 1985; Leyi Zheng leg.; from capsule of *Abutilonindicum* (NKUM) • 192♂♂183♀♀; Hainan, Sanya; alt. 10 m; 5–6 Apr. 1960; Suofu Li leg. (IZAS) • 26♂♂26♀♀; Hainan, Ledong; 11 Jun. 1960; Xuezhong Zhang leg. (IZAS) • 1♂; Hainan Nada; 27 Apr. 1954; Keren Huang leg. (IZAS).

**Figure 1.**
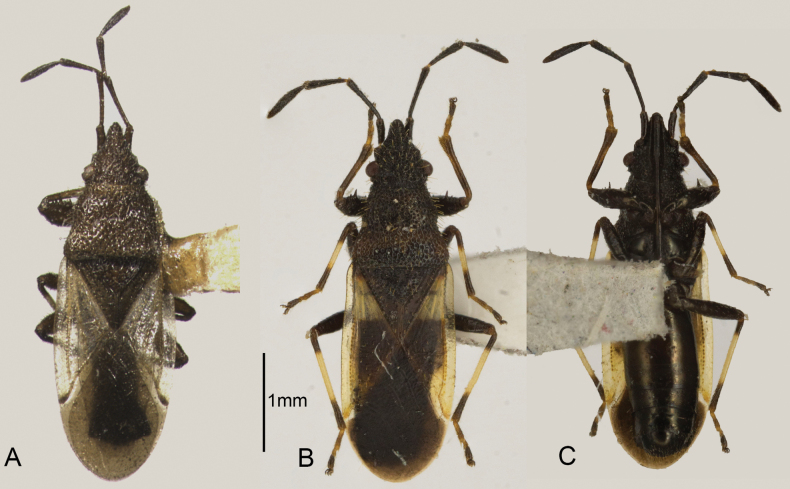
Dorsal and ventral view of *Oxycarenus* species sequenced **A***O.gossypii***B, C***O.bicolorheraldus*.

##### Redescription.

Body brown, densely punctate, with white decumbent, erect, and apically enlarged setae. Antennae dark brown. Rostrum extends past anterior margin of abdominal sternite III, up to abdominal sternite V in females. Bucculae yellowish white. Pronotum brown, often lighter at anterior margin and posterior half, densely covered with deep, large punctures, white erect, and apically enlarged setae mixed with decumbent setae; callus area slightly elevated, densely covered with large, dark brown setae. Lateral margins of pronotum slightly sinuate. Scutellum brown, evenly punctate, flattened except basal margin concave, peripherally covered with both decumbent and erect, apically enlarged setae. Clavus brown, with both types of setae mentioned above. Corium yellowish brown, with a conspicuous black spot at distal angle; sparse erect setae, apical margin straight. Membrane smoky brown. Thoracic sternum brown, posterior margins of metapleura pale. Ostiolar peritreme of metathoracic scent gland yellow. Supracoxal lobewhite. Femora dark brown; fore femora beneath with four spines; fore tibiae yellowish brown; mid and hind tibiae pale, both ends brown. Abdominal sterna reddish brown, smooth, impunctate, without erect setae. Male sternites VI and VII with posterior margin with two transverse combs of glandular setae on either side of median line. Female abdominal sterna III to IV fused; ovipositor reaching abdominal sternites V–VII, with sternites V–VII medially strongly narrowed, pushed forward towards base of abdomen.

**Figure 2.**
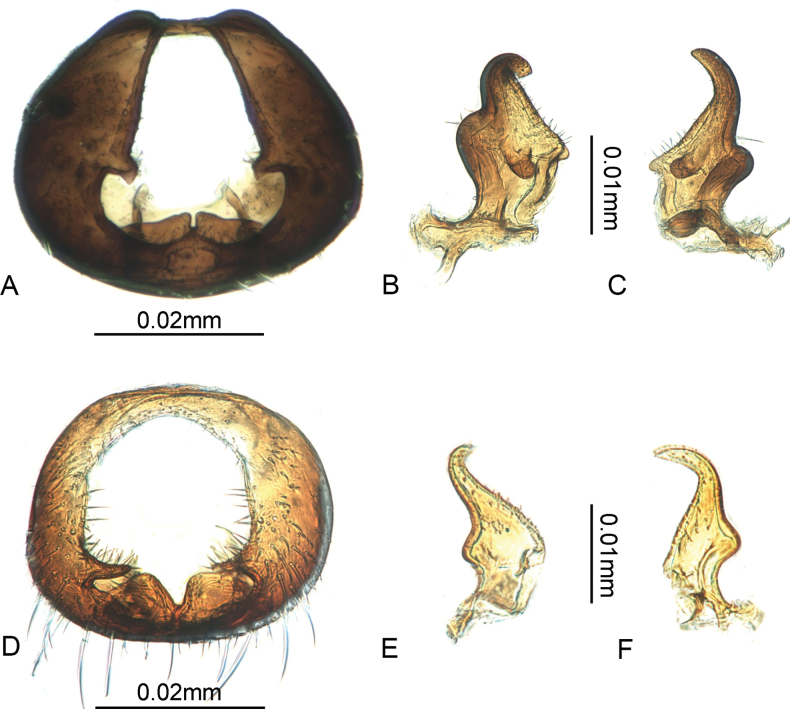
Genitalia of *Oxycarenus* species **A–C***O.gossypii***A** pygophore, posterodorsal view **B, C** left paramere, dorsal and ventral view **D–F***O.bicolorheraldus***D** pygophore, posterodorsal view **E, F** left paramere, dorsal and ventral view.

Pygophore: dorsal opening narrowly triangular (Fig. 2A); lateral projections in basal one third of pygophore openings, projecting obliquely posteriorly, tips truncate; distal margin of cup-like sclerite with a narrow, deep incision (Fig. 2A). Parameres (Fig. 2B, C) with basal shank relatively broad, about twice as wide as blade; outer projection rounded, inner projection more pointed from dorsal view; another finger-like inner projection present on inner side from ventral view.

***Measurements*** (in mm, *n* = 8). Body length 3.40–4.00, width 1.1–1.30. Head length 0.70–0.72, width across eyes 0.65–0.67; antennal segments I–IV length: 0.27–0.29: 0.56–0.58: 0.45–0.47: 0.52–0.54. Pronotum length 0.78–0.80, width of anterior margin 0.52–0.54, width of posterior margin 1.00–1.02; scutellum length 0.34–0.36, width 0.52–0.54. Distance of apex clavus–apex corium 0.60–0.62; distance of apex corium–apex membrane 0.72–0.74.

##### Distribution.

China (Hainan, Yunnan, Taiwan); Vietnam.

##### Remarks.

On review of descriptions and figures, we discovered that *Oxycarenusgossypii* was erroneously identified as *Oxycarenuslaetus* (Kirby, 1891) in the study by Zheng and Zou (1981). However, distinct differences exist between these species: the clavus appears brown in *O.gossypii*, whereas it is pale in *O.laetus*; the membrane presents a smoky golden-brown hue in *O.gossypii* (in contrast to the colorless and hyaline membrane of *O.laetus*); and, while the corium of *O.gossypii* is pale or slightly smoky except at the base, it consistently remains pale in *O.laetus*.

#### ﻿ Oxycarenus bicolor heraldus

Taxon classificationAnimaliaHemipteraOxycarenidae

Distant, 1904

8BDACA5D-4334-58B3-A37D-FEEF2B50885F

Figs 1B, C , 2D–F

Oxycarenus heraldus : Distant 1904: 44.
Oxycarenus bicolor variety heraldus : Bergroth 1918: 73.
Oxycarenus bicolor heraldus : Slater 1964: 670.
Oxycarenus lugubris : Zheng and Zou 1981: 97. Misidentification.

##### Type material examined

**(digital photograph). *Lectotype***: Burma • ♀; Carin Chebà [pr] / 900–1100 m [pr] / L. Fea V XII-88 [pr] // *heraldus* [hw] / Dist. [hw] // Distant Coll. / 1911–383 // Type [pr, red] // SYN/ TYPE [pr, blue] // *Oxycarenus* / *heraldus* / Distant, 1904: 44 [pr] / BMNH(E) / 1340705 [pr] (BMNH).

***Paralectotype***: same information except: BMNH(E) / 1340706 [pr].

##### Other material examined.

China • 2♀♀; Gansu, Wen county, Fanba; 30 Jul. 1988; collected from capsule of *Abutilontheophrasti* (NKUM) • 20♂♂15♀♀; Jiangsu, Nanjing, Laoshan; 20 Jun. 2021; collected from capsule of *Hibiscusmutabilis* (NJFU) • 6♂♂7♀♀; Sichuan, Qingchengshan; 16 Aug. 1956; Leyi Zheng leg. (NKUM) • 20♂♂25♀♀; Yunnan, Dali, Cangshan; 19 Aug. 2006; Zhonghua Fan leg. (NKUM) • 300♂♂242♀♀; Yunnan, Menglong, Banna, Mengsong, alt. 1600 m; 23 Apr. 1958; Xvwu Meng leg. (IZAS) • 40♂♂32♀♀; Yunnan, Pingbian; alt. 1300 m; 22 Jun. 1956; Keren Huang leg. (MSIE).

##### Redescription.

Head dark, blackish brown or black, densely coarsely punctate, with white, flat, decumbent setae and sparser erect, apically enlarged, long setae. Antennae dark, blackish brown or black, with segment I extending to tip of clypeus. Head ventrally densely covered with silvery-white, flat setae. Rostrum extends to hind coxae or middle of abdominal sternite III. Bucculae dark. Pronotum brown with a black transverse stripe at callus area. Covered with coarse punctures and sparsely erect and apically enlarged long setae, with slightly sinuate lateral margins. Scutellum dark blackish brown or black, similar setae to pronutum, punctuated, with a sunken base and a slightly elevated middle. Clavus dark brown to blackish brown, possessing three lines of punctures, with middle row incomplete. Corium with exocorium, basal 1/3 of inner corium, and distal angle yellowish white, about middle 1/3 of inner corium blackish brown, not reaching exocorium; sometimes, extreme distal angles of corium slightly darkened, but not with obvious small black spots; distal margin of corium straight; clavus and corium with sparse pale erect setae. Membrane dark blackish brown, with basal margin adjoining distal margin of corium narrowly white. Head and prothorax ventrally densely covered with silvery-white, decumbent setae; thoracic sternites and pleurae black or dark blackish brown, glossy, except supracoxal lobe and posterior margin of metapleura pale; ostiolar peritreme of metathoracic scent gland strongly protruding, basally brown and distally yellow. Femora blackish brown, slightly thickened; fore femora with four spines; tibiae yellow with both ends dark blackish brown, and fore tibiae darker. Abdomen reddish brown to blackish brown. Posterior margin of sternites VI and VII in males with two conspicuous transverse combs of glandular setae on either side of median line. Female abdominal sterna III–IV fused; ovipositor reaching abdominal sternites V–VII, with sternites V–VII medially strongly narrowed and pushed forward towards abdominal sternites V.

Pygophore: dorsal opening broadly rounded; lateral projections finger-like, slightly inclined posteriorly and internally; distal margin of cup-like sclerite bifurcate (Fig. 2D). Parameres with blade falcate and curved; outer projection rounded; inner projection projecting dorsoventrally, more square (Fig. 2E, F).

***Measurements*** (in mm, *n* = 8). Body length 3.80–4.30, width 1.10–1.40. Head length 0.71–0.73, width across eyes 0.72–0.73; antennal segments I–IV length: 0.28–0.30: 0.61–0.63: 0.47–0.49: 0.58–0.60. Pronotum length 0.83–0.85, width of anterior margin 0.58–0.60, width of posterior margin 1.10–1.11; scutellum length 0.41–0.43, width 0.54–0.55. Distance of apex clavus–apex corium 0.89–0.90; distance of apex corium–apex membrane 0.67–0.69.

##### Distribution.

China (Gansu, Jiangsu, Hubei, Sichuan, Yunnan); Burma.

##### Remarks.

The specific status of *Oxycarenusheraldus* Distant, 1904 was previously reduced to *Oxycarenusbicolorvar.heraldus* by Bergroth (1918), and later treated as subspecies *Oxycarenusbicolorheraldus* by Slater (1964).

*Oxycarenusbicolorheraldus* shares similar coloration with *Oxycarenusbicolorbicolor*, but there are notable differences. Unlike *Oxycarenusbicolorbicolor*, the brown spots on the hemelytra of *Oxycarenusbicolorheraldus* do not reach the exocorium (the brown spots on the hemelytra extend to the lateral margin of the corium in *O.bicolorbicolor*). Furthermore, the body size of *O.bicolorheraldus* is larger (3.80–4.30 mm) compared to *O.bicolor* (which is smaller, approximately 3.0–3.4 mm), and while the postero-lateral angles of the corium in *O.bicolorheraldus* may be slightly darkened, but they lack the distinct small black spots that are present in *O.bicolorbicolor*.

*Oxycarenusbicolorheraldus* is a common species in China, but it has long been misidentified as *Oxycarenuslugubris* (Motschulsky, 1859) (Zheng and Zou 1981). In comparison with *O.lugubris*, the pronotum of *O.bicolorheraldus* is brown with a black transverse stripe, whereas in *O.lugubris*, it is entirely black. Furthermore, only the middle 1/3 of the inner corium is blackish brown in *O.bicolorheraldus*, with the basal membrane narrowly white, while the distal 2/3 of the inner corium is entirely black, and the base of the membrane is also black in *O.lugubris*. Although both the species are distributed in China, *O.lugubris* has only been recorded from Taiwan and Hong Kong according to the data available on the iNaturalist website.

### ﻿Genome structure and base composition

**Figure 3.**
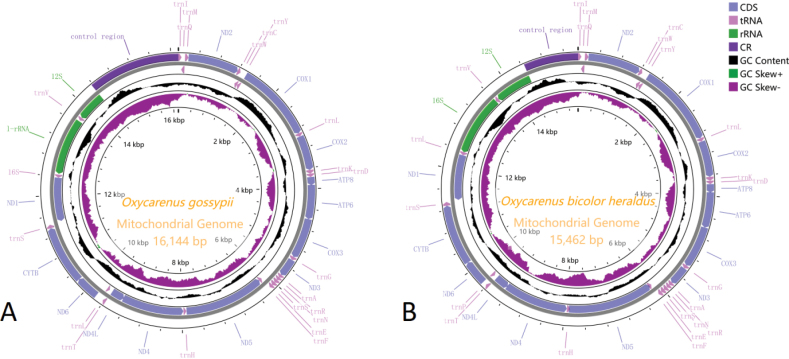
Circular map of the complete mitogenome of *Oxycarenus* species **A***O.gossypii***B***O.bicolorheraldus*. Different colors indicate different types of genes and regions. Genes in the outer circle are located on the J-strand, and genes in the inner circle are located on the N-strand.

We have sequenced and annotated the complete mitogenomes of *O.gossypii* and *O.bicolorheraldus*, which were 16,144 bp and 15,462 bp in length, respectively (Table 1). These mitogenome sequences consist of the 37 typical insect mitochondrial genes, including 13 protein-coding genes (PCGs), 22 transfer RNA genes (tRNAs), and two ribosomal RNA genes (rRNAs), along with an AT-rich region known as the control region (CR), forming a double-stranded ring structure (Fig. 3). The N-strand encodes 14 genes, while the J-strand encodes 23 genes, consistent with the mitochondrial gene arrangement observed in known Lygaeoidea species and the classical insect *Drosophilayakuba* (Burla, 1954) (Clary and Wolstenholme 1985; Hua et al. 2008; Küechler et al. 2010; Cao et al. 2020).

The nucleotide composition of the *O.gossypii* mitogenome was as follows: A = 41.35%, T = 32.82%, C = 15.33%, and G = 10.50%, while that of *O.bicolorheraldus* was A = 40.86%, T = 33.11%, C = 15.68%, and G = 10.35%. Both mitogenomes exhibited a high AT content, with *O.gossypii* at 74.17% and *O.bicolorheraldus* at 73.97%. Additionally, both mitogenomes displayed a slightly positive AT-skew (0.11 and 0.10) and a negative GC-skew (−0.18 and −0.20), indicating a bias towards A and T nucleotides. The study identified 15 gaps in the two mitogenome sequences, ranging from 1 bp to 22 bp, with the longest intergenic spacer being 22 bp, found between *rrnL* and *trnV* in *O.gossypii* (Table 2). Moreover, there were 25 overlapping gene regions, with lengths ranging from 1 bp to 24 bp, and the longest overlap of 24 bp was observed between *nad5* and *trnH* in *O.bicolorheraldus* (Table 3).

**Table 2.** Mitochondrial composition of *Oxycarenusgossypii*.

Name	Direction	Position From	Position To	Length (bp)	Intergenic nucleotides	Start/Stop Codons
*trnI*	J	1	62	62	3	
*trnQ*	N	60	128	69	-1	
*trnM*	J	130	197	68	0	
*nad2*	J	198	1187	990	2	ATA/TAA
*trnW*	J	1186	1248	63	8	
*trnC*	N	1241	1302	62	-1	
*trnY*	N	1304	1364	62	-1	
*cox1*	J	1366	2899	1534	0	TTG/T - -
*trnL2*	J	2900	2964	65	0	
*cox2*	J	2965	3640	676	0	ATA/T - -
*trnK*	J	3641	3711	71	0	
*trnD*	J	3712	3777	66	0	
*atp8*	J	3778	3936	159	7	ATT/TAA
*atp6*	J	3930	4595	666	1	ATG/TAA
*cox3*	J	4595	5381	787	0	ATG/TAA
*trnG*	J	5382	5447	66	0	
*nad3*	J	5448	5801	354	0	ATA/TAA
*trnA*	J	5802	5864	63	0	
*trnR*	J	5865	5927	63	0	
*trnN*	J	5928	5995	68	1	
*trnS1*	J	5995	6063	69	1	
*trnE*	J	6063	6127	65	0	
*trnF*	N	6128	6190	63	1	
*nad5*	N	6190	7899	1710	-3	ATA/TAA
*trnH*	N	7903	7964	62	-2	
*nad4*	N	7967	9286	1320	7	ATG/TAA
*nad4l*	N	9280	9558	279	-5	ATA/TAA
*trnT*	J	9564	9625	62	0	
*trnP*	N	9626	9684	59	4	
*nad6*	J	9781	10236	456	1	ATA/TAA
*cytb*	J	10236	11370	1135	0	ATG/T - -
*trnS2*	J	11371	11439	69	-16	
*nad1*	N	11456	12379	924	0	ATT/TAA
*trnL1*	N	12380	12445	66	0	
*rrnL*	N	12464	13671	1208	-22	
*trnV*	N	13694	13690	67	-4	
*rrnS*	N	13765	14372	608	0	

**Table 3.** Mitochondrial composition of *Oxycarenusbicolorheraldus*.

Name	Direction	Position From	Position To	Length (bp)	Intergenic nucleotides	Start/Stop Codons
*trnI*	J	1	62	62	3	
*trnQ*	N	60	128	69	1	
*trnM*	J	128	195	68	0	
*nad2*	J	196	1183	988	1	ATA/TAA
*trnW*	J	1185	1246	62	8	
*trnC*	N	1239	1300	62	0	
*trnY*	N	1301	1363	63	-1	
*cox1*	J	1365	2898	1581	0	TTG/T - -
*trnL2*	J	2899	2963	65	0	
*cox2*	J	2964	3639	699	0	ATA/T - -
*trnK*	J	3640	3711	73	0	
*trnD*	J	3712	3774	63	0	
*atp8*	J	3775	3933	159	7	ATA/TAA
*atp6*	J	3927	4592	666	1	ATG/TAA
*cox3*	J	4592	5378	790	0	ATG/T - -
*trnG*	J	5379	5443	65	0	
*nad3*	J	5444	5795	354	-1	ATT/TAG
*trnA*	J	5797	5859	63	0	
*trnR*	J	5860	5922	65	-1	
*trnN*	J	5924	5989	66	1	
*trnS1*	J	5989	6057	69	1	
*trnE*	J	6057	6122	65	0	
*trnF*	N	6123	6187	63	20	
*nad5*	N	6168	7922	1714	24	ATA/TAA
*trnH*	N	7899	7960	70	-2	
*nad4*	N	7963	9282	1320	7	ATG/TAA
*nad4l*	N	9276	9557	282	-2	ATT/TAA
*trnT*	J	9560	9621	62	0	
*trnP*	N	9622	9684	63	3	
*nad6*	J	9692	10153	462	1	ATT/TAA
*cytb*	J	10153	11289	1137	2	ATG/TAG
*trnS2*	J	11288	11358	71	-17	
*nad1*	N	11376	12298	960	0	ATA/TAA
*trnL1*	N	12299	12365	67	0	
*rrnL*	N	12366	13611	1253	0	
*trnV*	N	13612	13678	67	-1	
*rrnS*	N	13680	14453	802	0	

### ﻿Protein-coding genes

The concatenated length of the 13 protein-coding genes (PCGs) of *O.gossypii* was 10,990 bp, encoding 3,663 amino acid residues. Similarly, the concatenated length of the 13 PCGs of *O.bicolorheraldus* was 11,112 bp, encoding 3,702 amino acids. Both species share the same arrangement in their mitochondrial genomes. The majority of PCGs initiate translation using the start codon ATN, except for *cox1*, which starts with TTG. There are three types of stop codons: TAA, TAG, and an incomplete stop codon T that is completed by the addition of 3′A residues to the mRNA.

**Figure 4.**
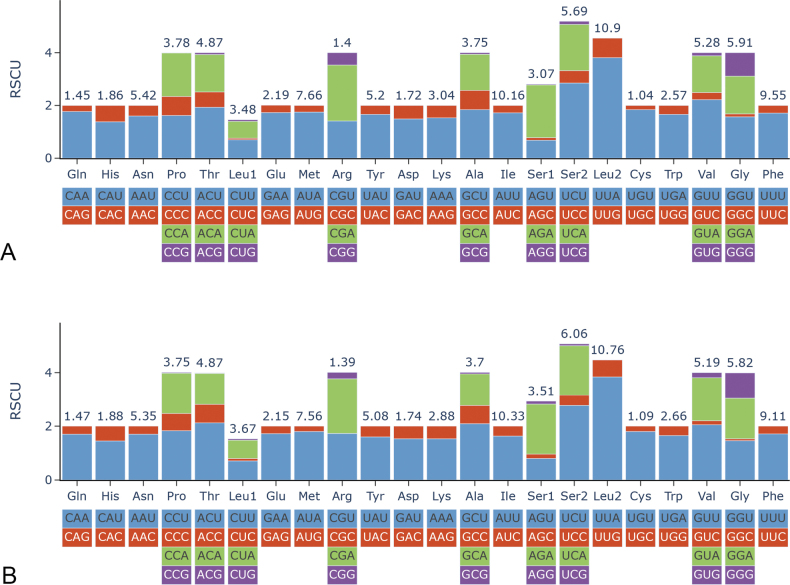
RSCU values of *Oxycarenus* species **A***O.gossypii***B***O.bicolorheraldus*. The ordinate represents the RSCU (the number of times a certain synonymous codon is used/the average number of times that all codons encoding the amino acid are used). The abscissa represents different amino acids. The number above the bar graph represents the ratio of amino acids (number of certain amino acids/total number of all amino acids).

**Figure 5.**
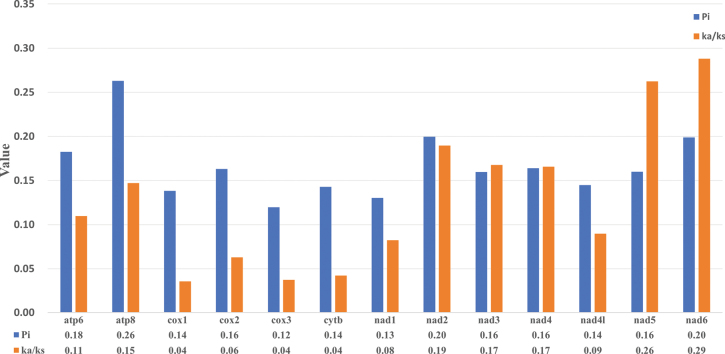
Nucleotide diversity (Pi) and nonsynonymous (Ka)/synonymous (Ks) mutation rate ratios of 13 PCGs of Oxycarenidae species (the Pi and Ka/Ks values of each PCG are shown under the gene name).

The Relative Synonymous Codon Usage (RSCU) of the two Oxycarenidae species was computed and depicted in Fig. 4. Among the codons utilized, CGA-Arg, GCU-Ala, UCU-Ser, UUA-Leu, and GUU-Val were the most frequently employed. Particularly, UUA emerged as the most preferred codon. Moreover, a pronounced bias toward A/T nucleotides was evident across the Protein-Coding Genes (PCGs). Nucleotide diversity (Pi) and the ratios of Ka/Ks for the two species were calculated based on the 13 PCGs, as illustrated in Fig. 5. Pi values ranged from 0.12 to 0.26, with the highest values observed in *atp8* and the lowest in *cox3*, underscoring *cox3*’s role as the most conserved gene in Oxycarenidae. All Ka/Ks ratios were below 1, varying from 0.04 to 0.29, indicative of purifying selection acting on the genes. Particularly noteworthy was *nad6*’s highest Ka/Ks values, suggesting rapid evolution, while *cox1* and *cytb* exhibited the slowest evolution, with the lowest values.

### ﻿RNA

The rRNA genes were positioned between the AT-rich region and *trnL1*, separated by *trnV*. Their total length ranged from 1816 bp to 1840 bp. In both species, the collective length of the 22 tRNA genes was 1433 bp, with individual tRNA genes varying from 61 bp to 71 bp. Notably, eight tRNA genes were encoded on the N-strand, while the remaining 14 genes were encoded on the J-strand, consistent with previous findings (Bernt et al. 2013; Cao et al. 2020).

Most tRNA genes exhibited a typical cloverleaf secondary structure, featuring a TΨC arm, an amino acid acceptor arm, an anticodon arm, and a dihydrouridine arm. However, an exception was observed in *trnS1*, where the dihydrouridine arm was absent in *O.gossypii*, forming a loop. Additionally, *trnS1* of *O.bicolorheraldus* displayed an atypical cloverleaf structure, as depicted in Suppl. material 1, a pattern also observed in other species (Zhao et al. 2018).

### ﻿Phylogenetic analysis

**Figure 6.**
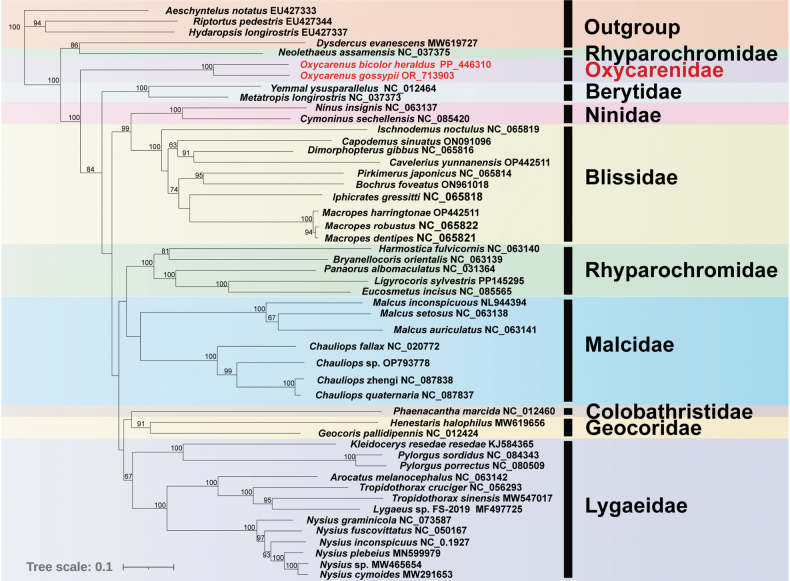
Phylogenetic tree of Lygaeoidea inferred from ML based on 13 PCGs. The numbers on the branches show bootstrap values (values >60% are shown). Two Oxycarenidae species in this study are marked in red.

**Figure 7.**
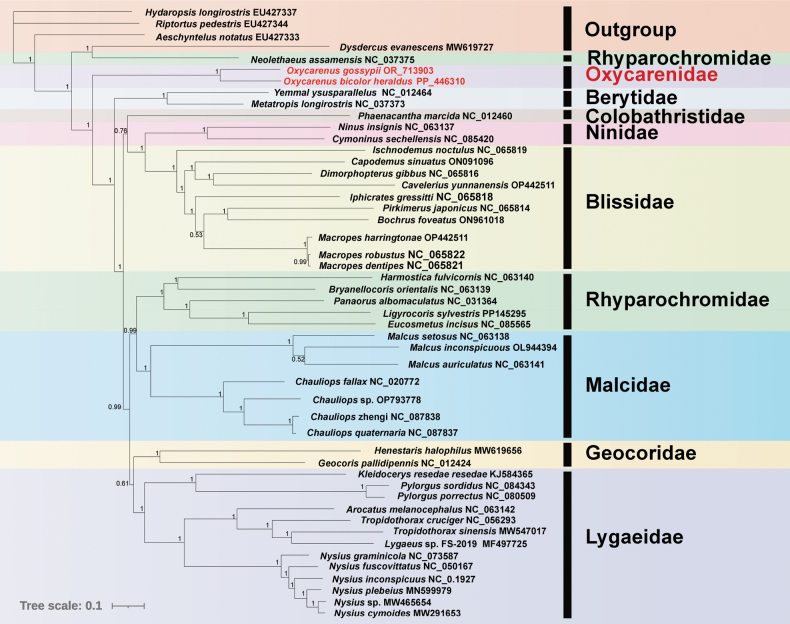
Phylogenetic tree of Lygaeoidea inferred from BI based on 13 PCGs. The numbers on the branches show posterior probabilities (values >0.50 are shown). Two Oxycarenidae species in this study are marked in red.

Phylogenetic relationships within Lygaeoidea were elucidated through the reconstruction of mitochondrial 13 PCGs using both BI and ML methods (Figs 6, 7). A total of 45 Lygaeoidea species were selected as the ingroup, with four species from Coreoidea and Pyrrhocoroidea serving as the outgroup. The resulting ML and BI trees exhibited slightly different topologies. Most families were consistently identified as monophyletic, except for Rhyparochromidae, which was paraphyletic. *Dysdercusevanescens* (Pyrrhocoroidea: Pyrrhocoridae) and *Neolethaeusassamensis* (Lygaeoidea: Rhyparochromidae) clustered together in both ML and BI trees (Figs 6, 7). The position of Colobathristidae proved to be unstable in the phylogenetic trees. In one instance, it clustered with Geocoridae with relatively low nodal support (Fig. 6), while another result indicated that Colobathristidae, Ninidae, and Blissidae formed a monophyletic group (Fig. 7). Furthermore, the two sequenced species of Oxycarenidae formed a single clade with a high support value.

## ﻿Discussion and conclusion

In this study, we redescribed two Oxycarenidae species: *Oxycarenusgossypii* and *O.bicolorheraldus*. We also detected misidentifications of two species in China. However, the sheer abundance and morphological similarities amongst oxycarenid species present challenges to providing an accurate morphology alone based classification.

The mitochondrial genomes of *O.gossypii* and *O.bicolorheraldus* were sequenced and analyzed, revealing a shared structural similarity. Both genomes exhibited a typical double-stranded ring structure housing 37 genes, including a non-coding control region. Remarkably, neither genome displayed any gene rearrangement, consistent with known genomic arrangements (Ding et al. 2023). The AT content significantly outweighed the CG content, showing a strong AT bias, a trait observed across various families within Pentatomomorpha (Guo and Yuan 2016). Our analysis of relative synonymous codon usage unveiled a prevalent preference for A/T codons, particularly at the termini of protein-coding genes, a phenomenon observed across all sequenced Pentatomomorpha (Hassanin et al. 2005; Guo and Yuan 2016). This nucleotide composition bias is believed to stem from a combination of mutational pressure and natural selection. The KA/KS analysis identified *cox1* and *cytb* as the most conserved genes, whereas *nad6* exhibited relatively higher evolutionary rates. Most protein-coding genes initiated translation using the start codon ATN, with the exception of *cox1* (TTG). Additionally, three types of stop codons were identified: TAA, TAG, and an incomplete stop codon T. While most tRNA molecules exhibited a typical cloverleaf structure, *trnS1* displayed an atypical cloverleaf structure in both species.

The monophyly of most families within Lygaeoidea was strongly supported, except for Rhyparochromidae, marking a deviation from Henry’s findings (1997). *Neolethaeusassamensis* (Lygaeoidea: Rhyparochromidae) clustering with *Dysdercusevanescens* (Pyrrhocoroidea: Pyrrhocoridae) in both ML and BI trees mirrored Gao and Dong’s (2023) results. The branches of Ischnorhynchinae, Lygaeinae, and Orsillinae formed a cohesive group designated as Lygaeidae, aligning with Gao and Dong’s findings (Gao and Dong 2023). The sister group relationship between *Henestarishalophilus* and *Geocorispallidipennis* supported Henry’s (1997) earlier assertion. However, the phylogenetic position of Colobathristidae remained unstable in our PCG-based tree, in contrast to Ye et al.’s (2022) findings. Moreover, our results did not support the hypothesis that Colobathristidae and Berytidae formed sister groups, nor did they form the “malcid line” with Malcidae and Cymidae as proposed by Henry (1997). The formation of a monophyletic group by Blissidae and Ninidae, excluding Berytidae, diverged from the inferred relationship based on 18S rRNA (Xie et al. 2005). However, our examination validated the hypothesis that the two sequenced Oxycarenidae species constituted a single clade, with the position of Oxycarenidae remaining stable in both ML and BI phylogenetic trees. While our findings enrich the structural information of mitochondrial genomes, a comprehensive discussion on the phylogenetic relationships within Lygaeoidea remains challenging. For a deeper understanding of their evolutionary history, it is imperative that more Lygaeoidea species are sequenced in future studies.

## Supplementary Material

XML Treatment for Oxycarenus gossypii

XML Treatment for Oxycarenus bicolor heraldus

## ﻿Additional information

### Author ORCIDs

Changjun Meng https://orcid.org/0009-0000-6968-8761

Suyan Cao https://orcid.org/0009-0008-4432-234X

Wen Dong https://orcid.org/0009-0004-6559-808X

Cuiqing Gao https://orcid.org/0000-0002-0177-5161

### Data availability

All of the data that support the findings of this study are available in the main text or Supplementary Information.

## Supplementary materials

Supplementary material 1 The predicted secondary cloverleaf structure for the *trnS1* of *Oxycarenusbicolorheraldus* and *O.gossypii* Data type docx https://binary.pensoft.net/file/1128432This dataset is made available under the Open Database License (http://opendatacommons.org/licenses/odbl/1.0/). The Open Database License (ODbL) is a license agreement intended to allow users to freely share, modify, and use this Dataset while maintaining this same freedom for others, provided that the original source and author(s) are credited.Changjun Meng, Suyan Cao, Wen Dong, Cuiqing Gao
