# Supplementary material for: ﻿The redescription and complete mitogenomes of two Oxycarenus species (Hemiptera, Oxycarenidae) and phylogenetic implications
Source: Zookeys. 2024 Sep 5;1211:231–50. doi: 10.3897/zookeys.1211.126013 (PMC11393489; doi:10.3897/zookeys.1211.126013)
Supplement: Supplementary material 1 — The predicted secondary cloverleaf structure for the trnS1 of Oxycarenusbicolorheraldus and O.gossypii [file zookeys-1211-231_article-126013__-s001.docx]

### Supplementary Materials


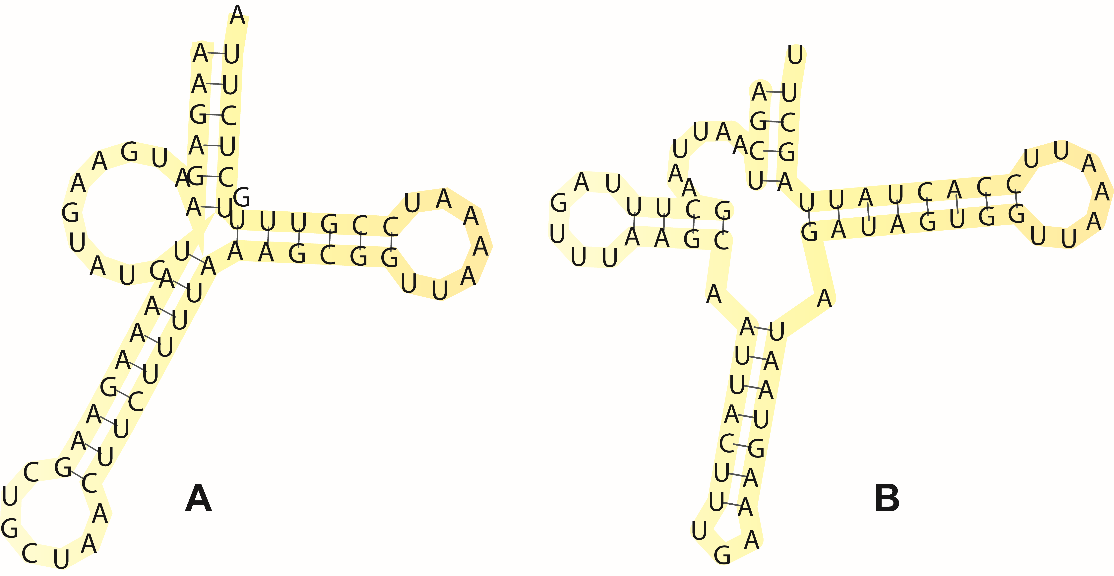


**Figure S1.** The predicted secondary cloverleaf structure for the *trnS1* of (**A**) *Oxycarenus* *bicolor heraldus* and (**B**) *O*. *gossypii.*
